# Electrochemical Behavior of Al(III) and Formation of Different Phases Al-Ni Alloys Deposits from LiCl-KCl-AlCl_3_ Molten Salt

**DOI:** 10.3390/ma11112113

**Published:** 2018-10-27

**Authors:** Yaru Peng, Zeng Chen, Ying Bai, Qingqing Pei, Wei Li, Chunli Diao, Xijin Li, Shengjun Li, Shaokang Dong

**Affiliations:** Henan Key Laboratory of Photovoltaic Materials, Henan University, Kaifeng 475001, China; 15896982806@163.com (P.Y.), ybai@henu.edu.cn (Y.B.), 15937815257@163.com (Q.P.), lw13598851727@163.com (W.L.), diaodcl@henu.edu.cn (C.D.), lxjzbj@126.com (X.L.), 18437970083@163.com (S.D.)

**Keywords:** molten salt, electrochemical behavior, Al-Ni alloys, phase composition, temperature

## Abstract

The electrochemical behaviors of Al(III) deposits on Ni substrates were investigated in LiCl-KCl-AlCl_3_ (2 wt.%) molten salts. Various electrochemical methods, including cyclic voltammetry (CV), square wave voltammetry (SWV), and open circuit chronopotentiometry (OCP) were used to explore the deposition processes of Al(III) on Ni substrates. Five kinds of Al-Ni alloys phase were firstly electrodeposited by the regulation of deposition potential form LiCl-KCl-AlCl_3_ (2 wt.%) molten salts at 753 K. The formation of Al-Ni alloys, such as AlNi_3_, Ni_5_Al_3_, AlNi, Al_3_Ni_2,_ and Al_3_Ni were confirmed by X-ray diffractometer (XRD) and the cross-section morphologies were investigated by scanning electron microscope (SEM). Meanwhile, it was found that the temperature of molten salt was another key parameter for the controlling of alloys phase. No Al-Ni alloys phase other than AlNi_3_ and Ni_5_Al_3_ could be deposited at 703 K.

## 1. Introduction

Aluminum alloys have been widely applied in the aerospace, automotive, and marine industries due to their low densities, high specific strength-to-weight ratios, and high electrical and thermal conductivities [1,2,3,4,5]. Al-Ni intermetallic alloys are the most important aluminum alloys with high melting points. For example, the melting points of AlNi and AlNi_3_ are 1638 °C and 1385 °C, respectively [6]. The Al-Ni intermetallic alloys also have high corrosion and oxidation resistances at elevated temperatures [2,7,8,9]. Thus, they have potential applications in high-temperature environments. 

In the past few decades, several methods have been explored to prepare Al-Ni intermetallic alloys. In general, there are two main ways to prepare Al-Ni intermetallic alloys. One avenue is based on the solid reaction at high temperature [2,5,10,11,12,13]. Sikka et al. reviewed the preparation method of AlNi_3_ and described their specific applications in heat-treating trays, radiant burner tubes, and transfer rolls for austenitizing furnaces [5]. Zhu et al. heated Ni/Al multilayer foil in a vacuum furnace to form AlNi, Al_3_Ni_2_ and Al_3_Ni alloys with different composite layers [11]. Ozdemir et al. repeated the similar process at 1050 °C under 150 MPa uniaxial pressures to yield single-phase AlNi alloys with very low porosity [2]. Minary et al. prepared Ni_3_Al by hot extrusion reaction synthesis, which could be carried out at low temperatures below 600 °C [12]. However, the high temperature solid state reaction method has some inherent disadvantages to produce Al-Ni alloys, such as inhomogeneity of chemical composition, expensive raw materials, high operation temperature, and large energy consumption. Poli et al. tried to improve the uniformity of Al-Ni alloy distribution through high energy ultrasonic dispersion in the synthesis process of AlNi intermetallic alloy. However, the ultrasonic dispersion had a limited propagation distance. The other way concerns the preparation of Al-Ni alloys through electrodeposition in non-aqueous solvents or metal salts [14,15]. The composition of Al-Ni alloys prepared with electrodeposition is well-distributed and the raw materials are metal salts, which are cheaper than the pure metal. Meanwhile, the deposition temperature is relatively lower than that of solid reaction method. For instance, Ali et al. electrodeposited Ni_3_Al on platinum and mild steel electrodes from aluminum chloride-N-(n-butyl) pyridinium chloride at room temperature [14]. However, there are five different phases of Al-Ni alloys (AlNi_3_, Ni_5_Al_3_, AlNi, Al_3_Ni_2_, and Al_3_Ni). It is a challenging work to prepare the five different phases of Al-Ni alloys through electrodeposit method. Jafarian et al. investigated the electrochemical nucleation and growth processes of Al(III) from molten salt NaCl-KCl-AlCl_3_ and proposed a random birth and deterministic growth of monolayers model after further study by impedance spectroscopy [15]. M. Ueda et al. obtained AlNi_3_ and Al_3_Ni through the electrochemical co-deposition of Al(III) and Ni(II) in the molten salt of NaCl-KCl-AlCl_3_-NiCl_2_ at 423 K. [16]. To this day, there is no report to prepare all the five phases of Al-Ni alloys through electrodeposition method. There are two main reasons for this status. One is the processing temperature is too low to form stable Al-Ni alloys phase in the usual electrolyte, such as room temperature ionic liquid, AlCl_3_-NaCl or AlCl_3_-KCl molten salt. The ionic liquid might become unstable and the AlCl_3_ in the AlCl_3_-NaCl (or AlCl_3_-KCl) molten salt might become seriously volatile when the deposition temperature is higher than 423 K. The other reason is that it is difficult to separately control the electrodeposition rate of Al and Ni in the co-deposition process. Therefore, the element content of Al-Ni alloys can’t be regulated conveniently. In this project, a small amount of AlCl_3_ (2 wt.%) was added into the eutectic LiCl-KCl mixture to form the molten salt. The electrochemical reduction process of Al(III) on Ni substrates was investigated at 703 K and 753 K, respectively. It studied the effects of deposition potential and temperature on the phase of Al-Ni alloys. Five different phases of Al-Ni intermetallic alloys were firstly prepared through electrodeposition in molten salt. An efficient way was recommended to prepare different phases of Al-Ni alloys coated Ni metal, which provides a material basis for the development of non-corrosive Ni metal. 

## 2. Experimental Section

All reagents were of analytical grade (Shanghai Aladdin Bio-Chem Technology Co. LTD, Shanghai, China) and used without further purification. The eutectic molten salts based on LiCl and KCl (mole ratio, 58.5:41.5) were dried in a vacuum oven for 5 h at 473 K before use. The AlCl_3_ (2 wt.%) was then added to the mixture in a reaction container composed of high-purity alumina crucible placed under an argon atmosphere. The molten salts were pre-electrolyzed at −1.25 V (vs. Pt) for 5 h. Before the electrodeposition of Al-Ni alloys, Mo wire was used as the working electrode to remove impurities from the melting media at 753 K [17].

A three-electrode system was employed for the electrochemical studies. The working electrode was either a Mo wire (Φ 0.5 mm), a Ni wire (Φ 0.5 mm) or a Ni plate (10 mm × 5 mm × 0.2 mm), depending on the intended experiment. The working electrodes were carefully polished and cleaned before use. The employed auxiliary electrode was a graphite rod (Φ 6.0 mm), and the quasi-reference electrode was Pt wire (Φ 0.5 mm) [18]. The metal electrodes were firstly washed with a detailed description of the testing system that can be found in previously published report [19,20,21]. 

All the electrochemical measurements were performed on a Im6 electrochemical workstation (Zahner Co., Ltd., Kansas City, MO, USA). The crystalline phases of the samples were characterized by DX-2700 X-ray diffractometry (XRD) with monochromatized Cu *Kα* irradiation (λ = 0.154145 nm). The morphologies of the cross-section of the alloys were examined by field-emission scanning electron microscopy (FE-SEM, JSM-7001F).

## 3. Results and Discussion

### 3.1. Cyclic Voltammetry

**Figure 1:**
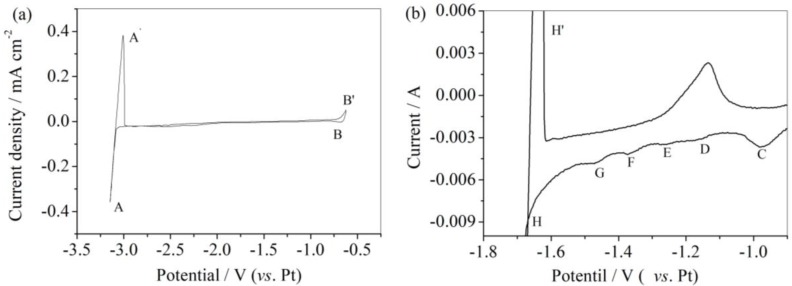
(**a**) Cyclic voltammogram of a Ni wire electrode from LiCl-KCl eutectic melt at 753 K and scan rate = 0.03 V s^−1^. (**b**) Cyclic voltammogram for a Ni sheet electrode in LiCl-KCl-AlCl_3_ (2 wt.%) melt at 753 K and scan rate = 0.03 V s^−1^.

The electrochemical behavior of the Ni wire electrode was firstly investigated in LiCl-KCl eutectic melt at 753 K and scan rate of 0.03 V s^−1^ (shown in Figure 1a). A sharp increase in the current density appeared at about −3.09 V (vs. Pt), corresponding to the reduction of Li^+^ ions. The observed anodic current peak (A’) was attributed to the dissolution of formed Li metal. The other redox peak (B/B’) was caused by dissolution of Ni wires. No other electrochemical processes were observed in the explored wide electrochemical windows, corresponding to Ni wire electrode studied in LiCl-KCl eutectic melt. This investigation indicated no reactions linked to impurities in LiCl-KCl eutectic melts. 

The cyclic voltammetry characterizations for Ni wire were also performed in LiCl-KCl-AlCl_3_ melt at 753 K and a scan rate of 0.03 V s^−1^, and the results are shown in Figure 1b. A series of new cathodic current peaks appeared before the deposition potential of Al subjected to cathodic potential sweep. These peaks were marked separately as C, D, E, F, G, and H. It is well known that Al-Ni alloys phase diagrams induce several Al-Ni intermetallic alloys at 753 K, including AlNi_3_, Ni_5_Al_3_, AlNi, Al_3_Ni_2_, and Al_3_Ni. Thus, the current peaks in Figure 1b were related to the formation of different Al-Ni intermetallic alloys. However, only three anodic current peaks were visible in the scanned potential range, which were less in numbers than the visible cathodic current peaks. This phenomenon might be induced by the low rate of some cathodic reactions [22]. However, it is still not clear which reaction processes corresponded to the observed three anodic current peaks. 

**Figure 2:**
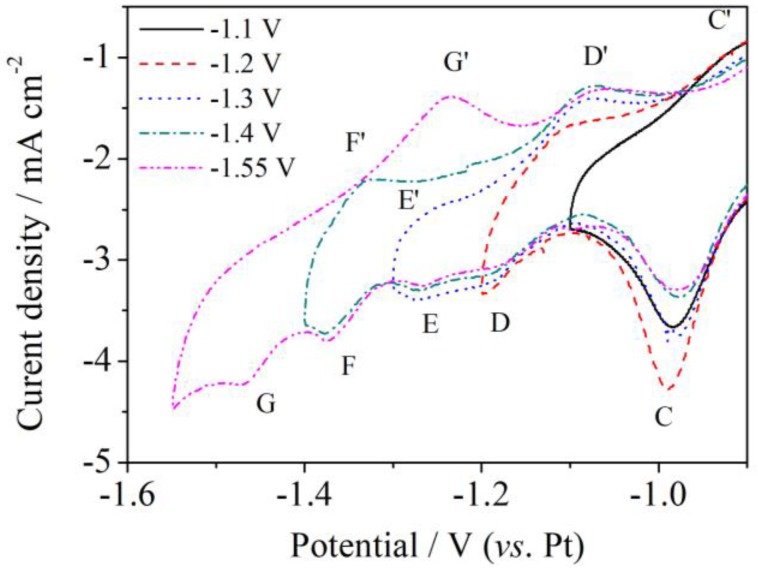
Cyclic voltammograms of a Ni wire electrode from LiCl-KCl-AlCl_3_ (2 wt.%) melt at 753 K and different inversion potentials at scan rate of 0.03 V s^−1^.

A series of cyclic voltammetry studies were carried out at different cathodic inversion potentials to identify the anodic processes shown in Figure 1b, and the data are gathered in Figure 2. The cathodic inversion potentials were respectively controlled at: −1.10 V, −1.20 V, −1.30 V, −1.40 V, −1.55 V, and −1.65 V (vs. Pt). The only anodic current peak (C’) obtained at the cathodic inversion potential of −1.10 V should correspond to the anodic dissolution of deposited alloys at ca. −0.98 V (vs. Pt) (peak C). Another anodic current peak (D’) appeared at −1.20 V (vs. Pt), confirming the existence of the cathodic process in D. A new cathodic current peak (E) also appeared at −1.30 V (vs. Pt) and a corresponding weak anode current peak (E’) appeared. Furthermore, a redox process (F/F’) was observed as the cathodic inversion potential was further shifted. The anodic current peak (F’) vanished when the cathodic inversion potential shifted to −1.55 V (vs. Pt) and another current peak (G’) appeared and linked to the anodic processes corresponding to the cathodic peaks (G). The anodic peak (F) might be covered by that of (G’) because the current peak was too large. Overall, the presence of five different cathodic peaks confirmed the formation of Al-Ni. 

### 3.2. Square Wave Voltammetry

**Figure 3:**
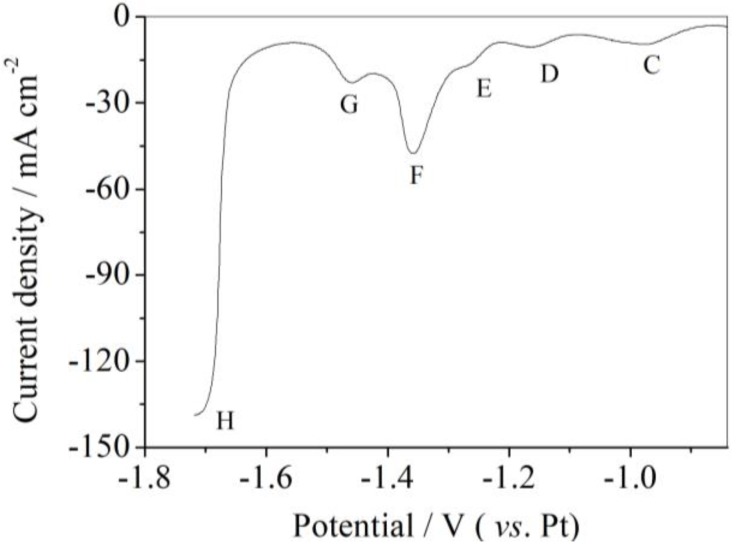
Square wave voltammogram of a Ni wire electrode in LiCl-KCl-AlCl_3_ (2 wt.%) melts plotted from −0.85 V to −1.72 V at 753 K.

Cyclic voltammetry showed six different cathodic reactions occurring during the cathodic scanning from −0.9 V to −1.7 V (vs. Pt). However, some of these cathodic reactions were too slow to detect. Hence, square wave voltammetry (SWV) was employed to gain further details of the redox processes because SWV has a much higher resolution than CV [23,24]. Thus, SWV was carried out from −0.85 V to −1.7 V (vs. Pt) in LiCl-KCl-AlCl_3_ (2 wt.%) melts at 753 K on Ni substrates, and the results are illustrated in Figure 3. The step potential adopted in this experiment was 1 mV and the pulse frequency was controlled to 20 Hz. Six significant current peaks appeared in the SWV curve. The deposition potential of each Al-Ni alloy might be obtained from the SWV curves. These potentials were determined as ca. −0.87 V, −1.11 V, −1.22 V, −1.30 V, −1.43 V and −1.67 V (vs. Pt). These peaks would correspond to the current peaks C, D, E, F, G and H (marked in the CV curves), respectively. Both the results of SWV and CV indicated that five types of Al-Ni alloys were formed during the electrodeposition of metal Al on Ni substrates. 

### 3.3. Open Circuit Chronopotentiometry

**Figure 4:**
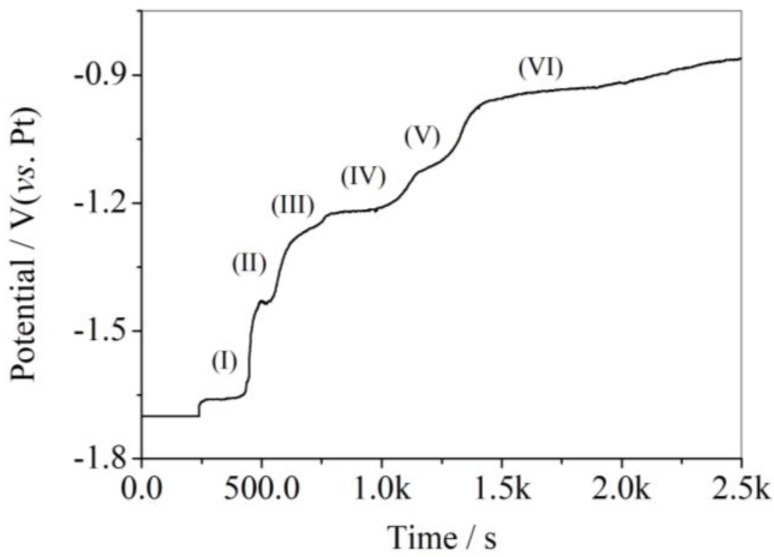
Open-circuit potential transient curves of a Ni sheet electrode after the application of an electrodeposition potential of −1.70 V for 4 min in LiCl-KCl-AlCl_3_ (2 wt.%) melts.

Open circuit chronopotentiometry (OCP) is another efficient method to explore the mechanism of electrodeposited processes from molten salts. For reversible and quasi-reversible processes, cathodic peaks should have corresponding anodic peaks. However, only two significant anodic current peaks in CV curves were visible. Thus, OCP was carried out on Ni plates from LiCl-KCl-AlCl_3_ (2 wt.%) melts at 753 K to further investigate the anodic processes of deposited Al-Ni alloys. A potentiostatic electrolysis was firstly performed on Ni plates from LiCl-KCl-AlCl_3_ (2 wt.%) melts at –1.70 V (vs. Pt) for 4 min. Thereafter, the potential was monitored as a function of time under open circuit conditions, and the collected data are given in Figure 4. In the open circuit chronopotentiogram, six potential plateaus were recorded at ca. −1.66 V, −1.43V, −1.27 V, −1.22 V, −1.12 V and −0.94 V, respectively. These potential plateaus were characteristic of dissolution processes of Al-Ni alloys. The first plateau at −1.66 V (vs. Pt) should be attributed to dissolved Al metal, and would correspond to the peak marked as (H’) in CVs. The other five potential plateaus should characterize the dissolution processes of Al_3_Ni, Al_3_Ni_2_, AlNi, Ni_5_Al_3_, and AlNi_3_. The thermodynamic properties of intermetallic, such as the Standard Gibbs free energies (Δ*G_f_^0^*), the partial molar Gibbs free energies (Δ*G_Al_*), and activities of Al (α_Al,Ni_), were also calculated from the OCP data (shown in the Supplementary Materials, Tables S1 and S2) [25,26]. 

### 3.4. Potentiostatic Electrolysis

To further explore the possible electrochemical reactions occurring during the reduction processes of Al(III) on Ni electrodes from LiCl-KCl-AlCl_3_ (2 wt.%) melts at 753 K, potentiostatic electrolysis was performed at different electrolysis potentials. According to CV, SWV, and OCP data, the electrolysis potentials were respectively set to −1.0 V, −1.17 V, −1.28 V, −1.37 V, and −1.47 V (vs. Pt). The deposited structures were characterized by XRD and the cross sections were visualized by SEM. Next, potentiostatic electrolysis was performed on Ni plates to identify the cathodic processes of Al(III) deposited from LiCl-KCl-AlCl_3_ (2 wt.%) melts. 

**Figure 5:**
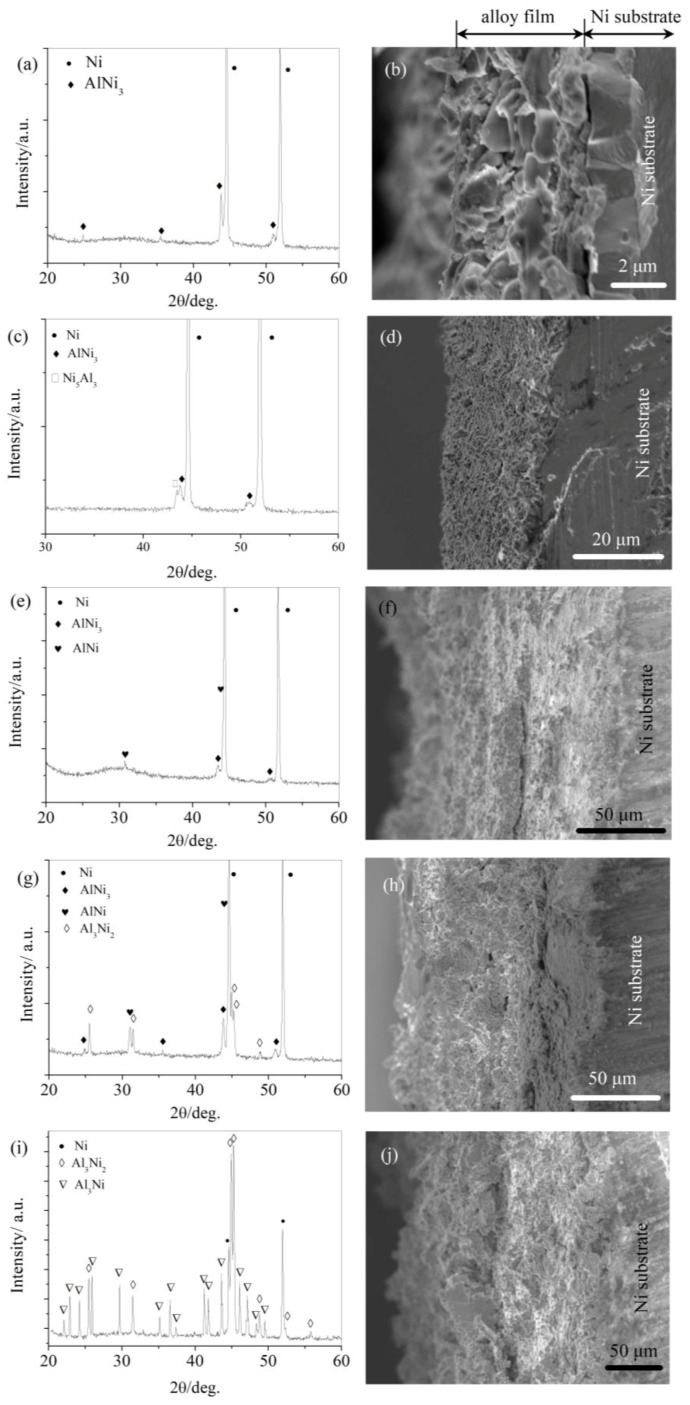
XRD and cross-sectional SEM image of samples obtained by potentiostatic electrolysis on Ni plate at different potentials from LiCl-KCl-AlCl_3_ (2 wt.%) melts at 753 K: (**a**,**b**) −1.0 V (vs. Pt) for 9 h; (**c**,**d**) −1.17 V (vs. Pt) for 12 h; (**e**,**f**) −1.28 V (vs. Pt) for 12 h; (**g**,**h**) −1.37 V (vs. Pt) for 12 h; and (**i**,**j**) −1.47 V (vs. Pt) for 12 h.

Figure 5 shows the XRD and SEM results, where it can be seen that AlNi_3_, Ni_5_Al_3_, AlNi, Al_3_Ni_2,_ and Al_3_Ni were deposited one after another as the cathodic deposition potential shifted from −1.0 V to −1.47 V (vs. Pt). This confirmed the occurrence of different Al(III) reduction processes on Ni substrates from LiCl-KCl-AlCl_3_ (2 wt.%) melt at different cathodic potentials. The reduction processes corresponding to the cathodic current peaks in Figure 3 might proceed as follows:

3Ni + Al(III) + 3e^−^ → AlNi_3_ (peak C) (1)

5/4AlNi_3_ + Al(III) + 3e^−^ → 3/4 Ni_5_Al_3_ (peak D) (2)

1/2Ni5Al3 + Al(III) + 3e^−^ → 5/2 AlNi (peak E) (3)

2Al Ni +Al(III) +3e^−^ → Al_3_Ni_2_ (peak F) (4)

1/3Al3 Ni2 + Al(III) +3e^−^ → 2/3 Al_3_Ni (peak G) (5)

The SEM of the cross sections showed compact Al-Ni alloy layers deposited on the Ni substrates at low cathodic potentials. The deposited alloy films depicted fine connections with the substrates. Alloy layers as thick as 100 μm could be deposited, indicating the potential of the proposed method for preparing Al-Ni alloys. However, the alloy layer contained some branches on the surface at −1.47 V (vs. Pt), perhaps caused by the non-uniformity of electrodeposition during the diffusion-limited processes. 

**Figure 6:**
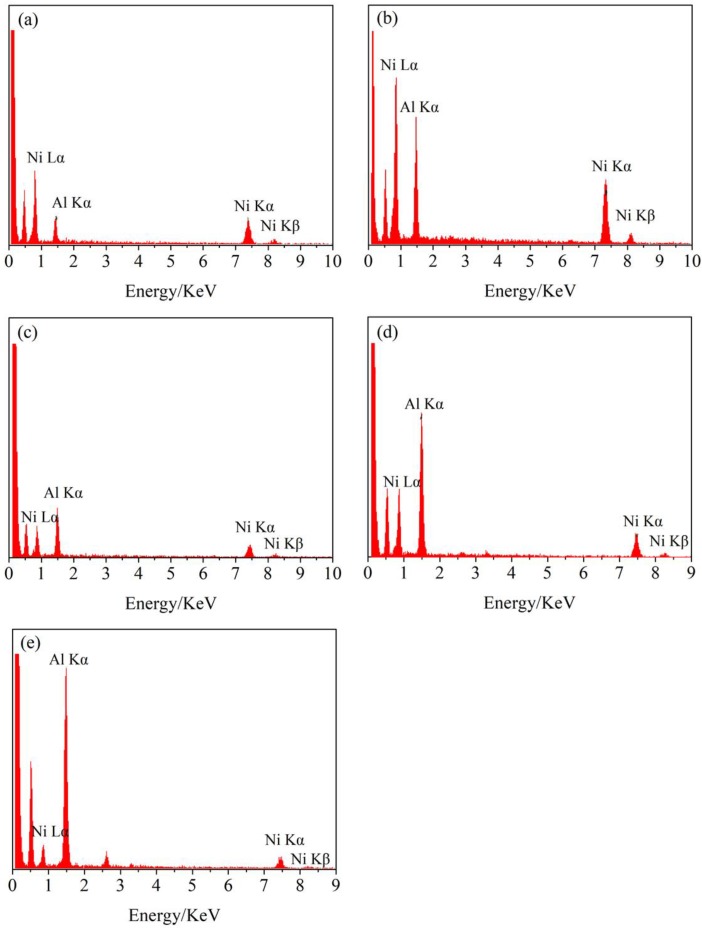
EDS of samples obtained by potentiostatic electrolysis on Ni plate at different potentials from LiCl-KCl-AlCl_3_ (2 wt.%) melts at 753 K, (**a**) −1.0 V (vs. Pt) for 9 h; (**b**) −1.17 V (vs. Pt) for 12 h; (**c**) −1.28 V (vs. Pt) for 12 h; (**d**) −1.37 V (vs. Pt) for 12 h, and (**e**) −1.47 V (vs. Pt) for 12 h.

EDS was also carried out to investigate the Al content at the surface of Ni substrate. The SEM-EDS analysis of samples electrodeposited at −1.0 V, −1.17 V, −1.28 V, −1.37 V, and −1.47 V (vs. Pt) was shown in Figure 6. The specific composition of the Al-Ni alloys was summarized in Table 1. The ratio between Al and Ni content is similar to the mole fraction of AlNi_3_, Ni_5_Al_3_, AlNi, Al_3_Ni_2,_ and Al_3_Ni, respectively. This result further indicated that different Al-Ni alloys phases could be prepared through the regulation of deposition potential. However, the obtained samples were not a pure phase of one kind of Al-Ni alloys when the deposition potential was more negative than −1.0 V. This inconsistent result might be related with the formation process of Al-Ni alloys in the electrodeposition process at high temperature. The formation process of Al-Ni alloys depends on the diffusion of Al atoms into the Ni substrate. Therefore, the composition of samples at the surface of Al-Ni alloys might be significantly different with that of the deeper section of Al-Ni alloys layer. Normally, the content of Al should run down steadily with the increase of depth in the Ni substrate. 

**Figure 7:**
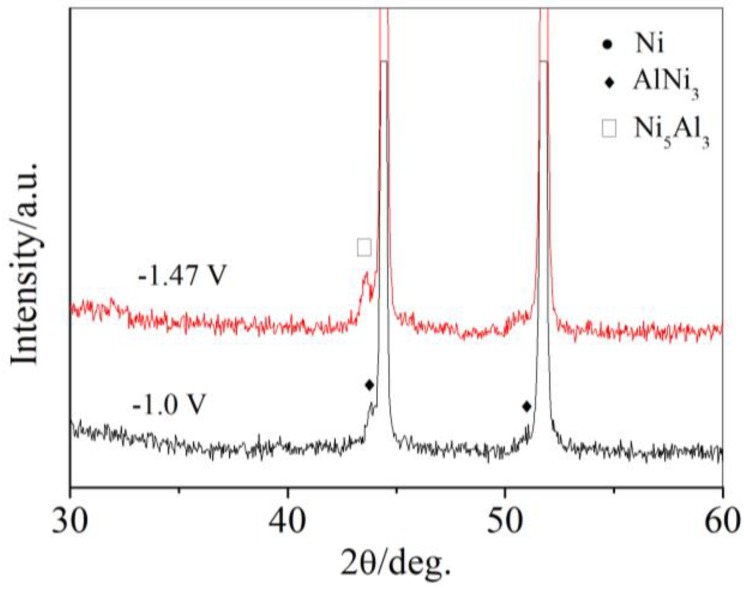
XRD of samples obtained by potentiostatic electrolysis on Ni plate at different potentials from LiCl-KCl-AlCl_3_ (2 wt.%) melts at 703 K.

The effects of molten salt temperature on the formation of Al-Ni alloys were also investigated. The XRD of the samples obtained at the deposition potential of −1.0 V and −1.47 V (vs. Pt) was shown in Figure 7. At the molten salt temperature of 703 K. At −1.0 V (vs. Pt), AlNi_3_ was also formed like the samples obtained at 753 K. The peak intensity was weakened. The samples obtained at −1.47 V (vs. Pt) showed significant differences from the samples obtained at 753 K. There was no other phase of Al-Ni alloys than Ni_5_Al_3_. This result indicated that the temperature of molten salt is the other key procedure condition other than potential for the formation of different phases of Al-Ni alloys. 

## 4. Conclusions

The electrochemical behaviors of Al(III) deposits on Ni substrates from LiCl-KCl-AlCl_3_ (2 wt.%) melt were investigated. Five different Al-Ni alloys were formed during the electrodeposition processes of Al(III), confirmed by SWV and OCP. Potentiostatic electrolysis experiments were also carried out to further confirm the electrodeposition processes of Al(III). Overall, AlNi_3_, Ni_5_Al_3_, AlNi, Al_3_Ni_2,_ and Al_3_Ni were firstly electrodeposited from molten salt at 753 K through the regulation of deposition potentials. The deposited Al-Ni alloys films showed compact morphologies with fine connections to the substrates, except the alloys deposited at −1.47 V (vs. Pt). Meanwhile, the temperature of molten salt was found to be the other key procedure condition other than potential for the formation of different phases of Al-Ni alloys. 

## Figures and Tables

**Table 1:** Al and Ni contents of the Al-Ni alloys obtained at different deposition potentials as determined by EDS.

Potential/V	Products	Al Content/at.%	Ni Content/at.%
−1.0 V	AlNi_3_	24.99	75.01
−1.17 V	AlNi_3_ + Ni_5_Al_3_	37.64	62.36
−1.3 V	AlNi_3_ + AlNi	51.55	48.55
−1.37 V	AlNi_3_ + AlNi + Al_3_Ni_2_	57.70	42.30
−1.47 V	Al_3_Ni_2_ + Al_3_Ni	74.22	25.78

## Supplementary Materials

The following are available online at http://www.mdpi.com/1996-1944/11/11/2113/s1, Table S1: Standard Gibbs free energies of formation for Al-Ni intermetallic compounds, Table S2: The partial molar Gibbs free energies and activities of Al in two-phase coexisting states at 753 K.
