# Supplementary material for: Electrochemical Behavior of Al(III) and Formation of Different Phases Al-Ni Alloys Deposits from LiCl-KCl-AlCl3 Molten Salt
Source: Materials (Basel). 2018 Oct 27;11(11):2113. doi: 10.3390/ma11112113 (PMC6265724; doi:10.3390/ma11112113)
Supplement: Supplementary file 1 [file materials-11-02113-s001.pdf]

Electronic Supplementary Information (ESI)

Electrochemical Behavior of Al(III) and Formation of Different Phases Al-Ni Alloys deposits from LiCl-KCl-$AlCl_3$ Molten Salt

Yaru Peng, Zeng Chen *, Ying Bai, Qingqing Pei, Wei Li, Chunli Diao, Xijin Li, Shengjun Li * and Shaokang Dong

Table S1. Standard Gibbs free energies of formation for Al-Ni intermetallic compounds.

Reaction	Equation for ΔG_f^0	$-\Delta G_f^0$ / kJ·mol⁻¹
Al(III) + 1/3 Al_3Ni_2 + 3 e = 2/3 Al_3Ni	$\frac{3}{2}(-3F\Delta E_6 + \frac{1}{3}\Delta G^0_{f,Al_3Ni_2})$	318.34
Al(III) + 2 AlNi + 3 e = Al_3Ni_2	$-3F\Delta E_5 + 2\Delta G^0_{f,AlNi}$	437.08
Al(III) + 1/2 Ni_5Al_3 + 3 e = 5/2 AlNi	$\frac{2}{5}(-3F\Delta E_4 + \frac{1}{2}\Delta G^0_{f,Ni_5Al_3})$	162.09
Al(III) + 5/4 $AlNi_3$ + 3 e = 3/4 Ni_5Al_3	$\frac{4}{3}(-3F\Delta E_5 + \frac{5}{4}\Delta G^0_{f,AlNi_3})$	555.75
Al(III) + 3 Ni + 3 e = $AlNi_3$	$-3F\Delta E_6$	208.41

Table S2. The partial molar Gibbs free energies and activities of Al in two-phase coexisting states at 753 K.

Plateau	E/V (vs. Pt)	ΔE/V (vs. Al(Ⅲ)/Al)	$-\Delta G_{Al}$/kJ·mol⁻¹	$\alpha_{Al,Ni}$
Ⅰ	-1.660 ± 0.003			
Ⅱ	-1.432 ± 0.003	0.23 (in the co-exiting Al_3Ni and Al_3Ni_2 phases)	66.58	2.41×10^{-5}
Ⅲ	-1.271 ± 0.005	0.39 (in the co-exiting Al_3Ni_2 and AlNi phases)	112.89	1.47×10^{-8}
Ⅳ	-1.219 ± 0.002	0.44 (in the co-exiting AlNi and Ni_5Al_3 phases)	127.36	1.46×10^{-9}
Ⅴ	-1.117 ± 0.006	0.54 (in the co-exiting Ni_5Al_3 and $AlNi_3$ phases)	156.31	1.43×10^{-11}
Ⅵ	-0.938 ± 0.006	0.72 (in the co-exiting $AlNi_3$ and Al phases)	208.41	3.48×10^{-15}
